# Dog-Assisted Therapy for Children and Adolescents With Fetal Alcohol Spectrum Disorders a Randomized Controlled Pilot Study

**DOI:** 10.3389/fpsyg.2020.01080

**Published:** 2020-05-26

**Authors:** Raquel Vidal, Laura Vidal, Francesc Ristol, Eva Domènec, Marta Segú, Cristina Vico, Núria Gomez-Barros, Josep Antoni Ramos-Quiroga

**Affiliations:** ^1^Department of Psychiatry, Hospital Universitari Vall d’Hebron, Barcelona, Spain; ^2^Group of Psychiatry, Mental Health and Addiction, Vall d’Hebron Research Institute, Barcelona, Spain; ^3^Biomedical Network Research Centre on Mental Health, Madrid, Spain; ^4^Department of Psychiatry and Legal Medicine, Universitat Autònoma de Barcelona, Barcelona, Spain; ^5^Centre de Terapia Assistida amb Cans, Barcelona, Spain; ^6^Fundación Probitas, Barcelona, Spain

**Keywords:** FASD, animal-assisted therapy, dog-assisted therapy, human-animal interactions, psychosocial treatments

## Abstract

**Objective:**

The rationale of this study was to evaluate the efficacy of dog-assisted therapy (DAT) combined with pharmacological treatment in children and adolescents with fetal alcohol spectrum disorder (FASD).

**Method:**

We conducted a randomized, rater-blinded, controlled pilot trial in a cohort of 33 children and adolescents with FASD. Participants were randomly assigned either to DAT group (*n* = 17) or Treatment as Usual (TAU control group) (*n* = 16).

**Results:**

Of the initial 39 participants enrolled, 33 completed treatment. A mixed-effects model analysis revealed that participants who were assigned to the DAT group experienced significantly improvements on social skills (SSIS-P social skills: *p* = 0.02, *d* = 0.8), reductions on externalizing symptoms (CBCL externalizing: *p* = 0.03; *d* = 0.56), and lower scores on FASD severity (CGI-S clinician: *p* = 0.001, *d* = 0.5).

**Conclusion:**

DAT is a promising adjunctive treatment for children and adolescents with FASD.

**Clinical Trial Registration:**

Dog-assisted therapy for children and adolescents with fetal alcohol spectrum disorders: a randomized controlled pilot study; http://clinicaltrials.gov/, identifier NCT04038164.

## Introduction

Prenatal alcohol exposure is one of the main preventable causes of birth defects and intellectual disability. The global prevalence of alcohol use during pregnancy is estimated to be 8–9% (Popova et al., 2017) and the estimated prevalence of fetal alcohol spectrum disorder (FASD) is between 1 and 5% (May et al., 2018). FASD is a complex condition which can be found in individuals prenatally exposed to alcohol. The spectrum of effects includes mild to severe cognitive, behavioral, physical, and sensory disabilities.

There are three distinct FASD diagnoses: complete fetal alcohol syndrome (FAS), partial FAS (pFAS), and alcohol-related neuro-developmental disorder (ARND) (Hoyme et al., 2016). FAS is characterized by a triad of features that includes (1) facial anomalies, (2) growth deficiency, deficient brain growth, abnormal morphogenesis, or abnormal neurophysiology, and (3) neurobehavioral impairment. pFAS is similar but with fewer or less severe physical features. In contrast, ARND, which accounts for most FASD case, has only neurobehavioral manifestations and no discernible physical characteristics. Across subtypes, patients with FASD present primary deficits that are the direct consequences of prenatal alcohol exposure; such as reduced memory, inattention, learning disabilities, difficulties in cause-effect thinking, poor social skills, and also difficulties in self-regulation. These primary deficits can lead to secondary difficulties such as school failure, legal problems, inappropriate sexual behavior, substance abuse, and difficulties in employment insertion. Furthermore, a 90% of the individuals with FASD present comorbid conditions, being attention deficit and hyperactivity disorder (ADHD) the most prevalent (Boseck et al., 2015; Weyrauch et al., 2017).

Despite consensus that patients with FASD need early intervention for children to prevent future adverse consequences, few therapies have demonstrated their efficacy (Hoagwood et al., 2017). According to the primary deficits on FASD, studies on psychological treatment have focused on social skills, emotional regulation, and behavioral problems. The most evidence-based treatments on FASD are the Alert Program based on emotional regulation (Wells et al., 2012; Nash et al., 2015, 2018) and the Friendship Program for social skills (O’Connor et al., 2006; Keil et al., 2010). However, to date, no studies have been published on the efficacy of dog-assisted therapy (DAT) in children and adolescents with FASD.

As interest in animal-assisted therapy (AAT) grows, there is an increasing need to differentiate informal activities from formal and professionally directed therapies. Despite a large body of evidence demonstrating the benefits of human-animal interaction, a significant number of studies are centered in animal-assisted activities (Calcaterra et al., 2015; Elmacı and Cevizci, 2015), which are not structured and without continued ATT. AAT is a goal-oriented, structured intervention provided by a certified professional working with a trained and certified animal which is an integral part of the treatment process.

Dog-assisted therapy is an ATT that has shown promising in children and adults with physical and mental diseases showing mainly reductions on anxiety and depression and improvements on social skills (Hoagwood et al., 2017; Hill et al., 2019; Jones et al., 2019; Wijker et al., 2019). However, most studies have relied on non-controlled designs, minimal use of randomization, small sample sizes, and limited descriptions of the intervention. There are several controlled studies on DAT in children that have proved their efficacy in other neurodevelopmental disorders such as ADHD and autism spectrum disorder (ASD). In ADHD, has obtained reduces in ADHD core symptoms, increases in self-steem (Schuck et al., 2018) and also in social skills (Schuck et al., 2015). In ASD, reductions on depressive symptoms and increases on social skills have been observed (Fung and Leung, 2014). Furthermore, DAT has also shown improvements in global functioning in adolescents with acute mental disorders (Stefanini et al., 2015). On the other hand, DAT has obtained benefits in pediatric oncology patients, obtaining reductions on stress (Silva and Osório, 2018; McCullough et al., 2018).

The rationale of the present study was to evaluate the efficacy of DAT combined with pharmacological treatment in children and adolescents with FASD in relation to its effects on social skills, on internalized and externalized symptomatology and on severity of FASD symptoms.

We hypothesized that, at the end of treatment, the DAT group combined with pharmacological treatment could exhibit more improvements on social skills, a reduction on internalized and externalized symptomatology and lower scores on severity of FASD symptoms, compared to the control group. It has been hypothesized that DAT could be an approach that might improve motivation to treatment and facilitate therapeutic alliance compared to other traditional interventions (Jones et al., 2019).

To our knowledge, this is the first study to evaluate the efficacy of DAT in children and adolescents with FASD.

## Materials and Methods

### Study Design

The design was a randomized, rater-blinded, controlled pilot trial. Participants were randomly assigned either to the DAT group (*n* = 17) or to the Treatment as Usual (TAU control group) (*n* = 16).

### Participants

Patients were recruited from the FASD Program in a university hospital in Barcelona, Spain. A psychologist and a psychiatrist were responsible for participant recruitment, they explained the potential consequences and study procedures to the patients. The inclusion criteria were as follows: patients diagnosed with FASD between 6 and 18 years (FAS, pFAS, or ARND) with or without comorbidities and with stabilized doses of medication for at least 2 months before the study. Patients with borderline IQ or intellectual disability were also included. Given the cognitive difficulties associated with the FASD we considered relevant to include this type of patients. Two patients do not accomplish inclusions criteria because they were not behaviorally stable so they required hospitalization, day hospital or more intensive treatments.

### Intervention

#### DAT Treatment Group

The DAT program comprised 12 manualized sessions and included two phases: (1) individual intervention (six sessions) and (2) group activity (six sessions). We used the CTAC Method (Center of Dog Assisted Therapy) (Domènec and Ristol, 2012). CTAC is a full-member of the International Association of Human-Animal Interaction Organizations (IAHAIO) which is the global association of organizations that engage in practice, research and/or education in animal assisted activity and animal assisted therapy. The sessions in the individual module were as follows: session 1, getting to know each other to determine the strengths and weaknesses of each patient; session 2, frustration tolerance and motivation; session 3, impulsivity management (self-control strategies, sequential thinking); session 4, emotional self-regulation (identifying emotional triggers and alarm signs); session 5, executive functions (planning, cause-effect thinking); session 6, review of the contents. The last six group sessions focused on social skills (criticism management, communication and cooperation, adaptive behavior, assertiveness training and empathy).

Patients participated in weekly sessions for about 3 months. Each session was conducted in a university hospital and lasted 45 min. In all sessions the patients participated without the presence of their parents and the groups were formed by 3–4 patients. Sessions included the participation of two certified therapy dogs, two DAT professionals and a psychologist. Dogs facilitated the achievement of the therapeutic goals set by the psychologist who conducted the intervention. All of the participating dogs were trained and tested to work with people, and their mental and physical health care was strictly monitored. CTAC meets the requirements of animal welfare according to the IAHAIO White Paper. Participants in this group were visited by their psychiatrist in order to monitor their adherence to medications.

#### TAU Control Group

Participants received their usual treatment. They were visited by their psychiatrist in order to monitor their adherence and continuation on medications as prescribed. Participants in the TAU group did not receive DAT sessions.

### Diagnostic and Outcome Measures

The diagnosis of FASD was established by experienced senior psychiatrists, a physical assessment was conducted by a geneticist and a neuropsychological evaluation carried out by a neuropsychologist. The diagnostic evaluation was administered only at the pre-treatment assessment.

The variables of the present study were as follows:

• Sociodemographic characteristics: patient’s characteristics (age and gender) and also parents’ characteristics (marital status and level of education) were assessed in the clinical interview during the diagnostic evaluation.
• FASD subtype: we used the Hoyme criteria that distinct three FASD subtypes: Complete FAS, pFAS, and ARND (Hoyme et al., 2016).
• Pharmacological treatment: medication was also registered and monitored in order to assess patient’s adherence and continuation on medication as prescribed by the psychiatrist.
• Comorbidity: to assess comorbidity we used the semi-structured K-SADS interview for children and adolescents aged under 16 (Kaufman et al., 1997), and the Structured Clinical Interview for DSM-IV Axis I and Axis II Disorders (SCID-I and SCID-II) for the evaluation of older adolescents (Spitzer et al., 1996; First et al., 1999).
• Intellectual Quotient: The Wechsler Intelligence Scale WISC-V or WAIS-V according to the patient’s age, was used in order to evaluate global cognitive capacity (Wechsler, 1981, 2003).

Outcome measures were administered at pre-treatment (T1 baseline time) and at the end of the treatment (T2).

• *Child Behavior Checklist (CBCL of Achenbach) parent version:* to assess Internalizing and Externalizing Symptoms in patients between 4 and 18 years. The CBCL is a 113 item scale which assesses withdrawn symptoms (e.g., acts to young, uncooperative), somatic complains (e.g., stomach aches, headaches), anxiety/depressive symptoms (e.g., feeling hurt, upset, nervous), thought problems (e.g., mind off, repeats acts), ADHD features (e.g., impulsive, acts too young), oppositional behavior (e.g., defiant, argues), and behavioral problems (e.g., no guilt, vandal). It has been validated in Spanish and has a reliability of 0.68–0.86 (Estrada et al., 2010). An internal consistency of 0.85 has been obtained in the study sample.
• *Social Skills Improvement System-Parent Form (SSIS-P)* (Spanish version): is a 79 items scale measuring social skills and problem behaviors in children and adolescents as reported by their parents. In the Social Skills domain, the subscales are communication, cooperation, assertion, responsibility, empathy, engagement, and self-control (e.g., expresses feeling when wronged, asks for help from adults, interacts well with other children, tries to comfort others). The problem behaviors domain includes internalizing and externalizing problems, bullying, hyperactivity/inattention, and autism spectrum (e.g. has temper tantrums, talks back to adults, lies or does not tell the truth, does things to make others feel scared). It has been validated in Spanish and has a reliability of 0.90 (Gresham and Elliott, 2008). An internal consistency of 0.86 has been obtained in the study sample.
• *Clinical Global Impression Scale for Severity*. The Clinical Global Impression Scale for Severity (CGI-S): is a 7-point scale (1 = normal, 2 = borderline mentally ill, 3 = mildly ill, 4 = moderately ill, 5 = markedly ill, 6 = severally ill, and 7 = extremely ill) (NIMH, 1985). We used the clinician version to evaluate FASD symptoms severity.

Parents completed all the measures, due to patients with FASD have poor self-awareness and the validity of their self-reports might be unclear (Rasmussen and Bisanz, 2009). Table 1 shows parents characteristics.

**TABLE 1:** Participant’s characteristics.

	**DAT group (*n* = 17)**		**TAU group (*n* = 16)**				
**Variable**	***n***	**%**	***n***	**%**	*****p*****	***X*^2^**	**Phi**
**Sex**							
Male	12	70.58	10	62.5	0.27	4.45	0.37
Female	5	29.41	6	37.5			
**FASD subtype**							
FAS	8	47.05	5	31.25	0.97	1.33	0.20
Partial FAS	6	35.29	5	31.25			
ARND	3	17.64	6	37.5			
**Comorbidity**							
ADHD + learning disorder	13	76.47	13	81.25	0.31	4.35	0.36
ADHD + ASD	3	17.64	1	6.25			
ADHD + ODD	1	5.88	2	12.5			
**Medication**							
Methylphenidate	10	58.82	12	75	0.45	1.55	0.21
Guanfacine	3	17.64	1	6.25			
Risperidone	2	11.76	2	12.5			
Atomoxetine	2	11.76	1	6.25			
**Parents characteristics**							
**Marital status**							
Married	11	64.70	12	70.58	0.34	6.25	0.44
Divorced	4	23.52	3	17.64			
Single	2	11.76	2	11.76			
**Level of education**							
Primary school	2	11.76	3	17.64	0.29	7.45	0.48
Secondary/baccalaureate	3	17.64	4	23.52			
Vocational	7	41.17	6	35.29			
University degree	5	29.41	4	23.52			

	**Mean**	**SD**	**Mean**	**SD**	***p***	***t***	**Cohen’s d**

Age	11.68	3.60	12.41	7.58	0.46	7.40	0.12
IQ	78.76	18.54	84.24	14.74	0.67	1.25	0.2
Parents age	47.56	4.62	48.45	6.23	0.52	6.45	0.16

### Procedure

The ethics committee of clinical investigation at the Hospital Universitari Vall d’Hebron approved the study before participant enrollment. The study was conducted between April 2018 and May 2019. During this period, 45 patients who were visited through the FASD unit were informed about the study, 2 did not met inclusion criteria and 4 declined to participate. Of the 39 participants enrolled, 33 completed treatment. Written informed consent was obtained from parents and written and informed assent was obtained from child and adolescents participants. After the pretreatment assessment, the data manager of the study randomized participants to the 2 treatment conditions using a computerized random number generator (SPSS version 20 software). The raters of the study were blinded to the intervention and were not involved in the trial except for interviewing the participants at pretreatment and post treatment assessments. Participants in the two groups were evaluated at the beginning of the study (T1) and at the end of the treatment (T2). Pre-test assessment was administered 1 week before the beginning of the intervention and the post-test assessment one week after the intervention that lasted 3 months (12 weekly sessions). Both the treatment group and the control group were run concurrently to control for effects of environmental factors (e.g., examinations or holiday periods).

### Statistical Analysis

Data were analyzed (using SPSS version 20) according to intent-to-treat principles using a last observation carried forward procedure. An analysis of variance for repeated measures was performed, analyzing group and time effect and its interaction effect. The treatment groups were compared on baseline characteristics using independent *t*-test and Chi-square analyses to verify the homogeneous distribution of variables between the groups. All reported results were significant at the 5% level. Cohen’s d was calculated to estimate the effect size of treatment outcomes.

## Results

### Sample Characteristics

Table 1 shows participant characteristics. No statistically significant differences between groups were detected with respect to clinical and baseline measures of the participants. No significant differences were detected between the two groups in the type and doses of medication.

### Program Completion Rate

**FIGURE 1:**
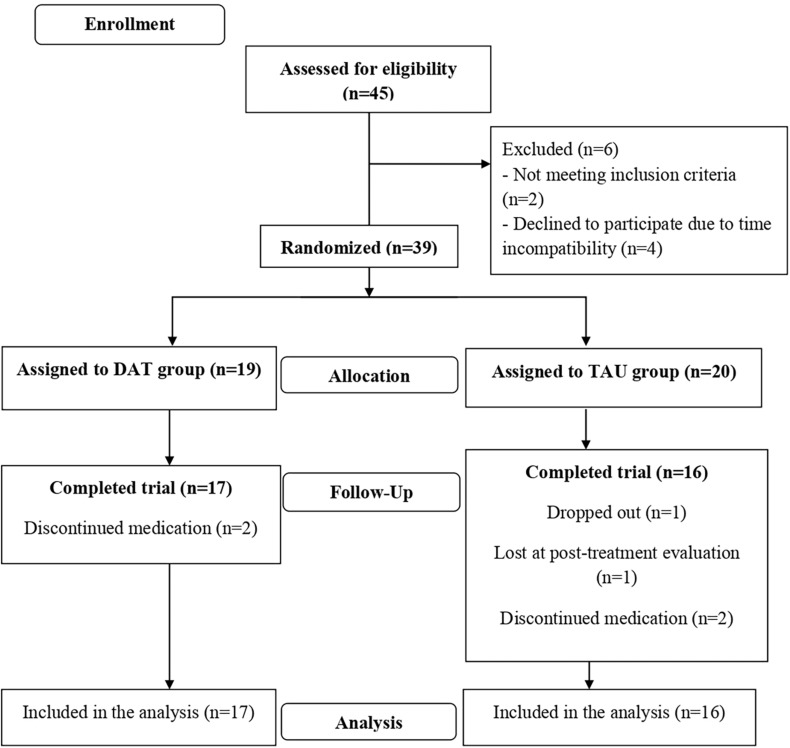
Flow diagram.

A flow diagram of the study is shown in Figure 1. Low dropout rates were observed, none of the participants in the DAT group dropped out. The treatment completion rate was high, all participants in the DAT group completed all sessions, and only two changed doses of medication prescribed by their psychiatrist.

Table 2 shows the results of the study.

**TABLE 2:** Outcome results.

**Measure**	**DAT group (*n* = 17)**		**TAU group (*n* = 16)**				
	**Baseline**	**Outcome**	**Baseline**	**Outcome**			
	**Mean (SD)**	**Mean (SD)**	**Mean (SD)**	**Mean (SD)**	**ES**	***p***	***F***
**CBCL externalizing**	16.48 (11.95)	11.12 (9.73)	16.95 (7.69)	16.25 (8.37)	0.56	0.03	12.35
ADHD	12.56 (5.56)	12.06 (5.28)	11.73 (5.23)	18.82 (3.73)	1.4	0.06	3.86
Opposition	4.5 (2.3)	4.5 (2.3)	5.45 (3.23)	5.27 (3.06)	0.2	0.06	8.32
Conduct problems	13.94 (9.37)	12.25 (8.45)	14.91 (6.15)	15.27 (6.15)	0.49	0.05	4.76
**CBCL internalizing**	16.88 (8.33)	14.62 (8.21)	16 (8.57)	13.73 (7.04)	0.11	0.2	3.51
Withdrawn	4.94 (3.29)	5.13 (3.91)	5.73 (3.55)	4.91 (2.84)	0.06	0.34	5.50
Somatic	1.31 (1.19)	0.94 (0.92)	1.0 (1.18)	0.82 (1.25)	0.1	0.24	2.85
Anxiety depression	11.6 (5.66)	9.31 (5.42)	9.45 (4.98)	8.27 (4.14)	0.2	0.16	4.74
Social problems	7.31 (3.45)	6.5 (3.42)	5.55 (4.2)	5.18 (3.31)	0.3	0.17	1.54
Thought problems	2.81 (2.48)	2.31 (2.49)	3.64 (3.82)	2.64 (3.58)	0.1	0.42	2.77
**SSIS-P social skills**							
Social skills	73.25 (17.08)	81.56 (16.58)	69.31 (13.86)	69.46 (13.51)	0.8	0.02	4.94
Problem behavior	36.75 (12.22)	33.88 (9.4)	40.15 (13.97)	41.77 (12.47)	0.7	0.1	3.59
**CSGI-S clinician**	3.76 (0.43)	3.18 (0.39)	3.44 (0.5)	3.44 (0.61)	0.5	0.01	6.51

*CBCL, Child Behavior Checklist; SSIS-P, Social Skills Improvement System-Parent Form; CGI-S, Clinical Global Impression Scale for Severity; p, significance on time-group Effect; ES, effect sizes, Cohen’s d.*

#### Internalizing and Externalizing Symptoms

A main effect of time was observed on externalizing symptoms [*F*(1.30) = 12.35, *p* = 0.001] and also a significant interaction on time × group was obtained; CBCL externalizing: [F(1.30) = 11.59, *p* = 0.03]. Medium effect sizes were found (*d* = 0.56). Results indicated a main effect of time on Internalizing symptoms [F(1.30) = 10.45, *p* = 0.001] but there was no significant interaction of time × group. These results indicated that both groups improved on internalizing symptoms from baseline regardless of the treatment condition they had been assigned.

#### Social Skills

A main effect on time was observed on social skills [F(1.30) = 15.54, *p* = 0.001] and an interaction time × group was obtained on social skills being the DAT group the one who improved more these abilities [F(1.30) = 13.82, *p* = 0.02]. Large effect sizes were found (SSIS-P social skills: *d* = 0.8). However, no interaction of time × group was observed on reductions on the problem behavior subscale in relation to social skills.

#### Severity of FASD Symptoms

A main effect of time was observed on the severity of FASD symptoms [F(1.30) = 12.549, *p* = 0.001] and also a main effect on time × group interaction, FASD severity decreased significantly more in the DAT group [F(1.30) = 16.54, *p* = 0.001]. Medium effect sizes were found (CGI-S clinician: *d* = 0.5).

### Moderators of Change

Clinical and demographic characteristics were included in the mixed-effect model to explore possible interactions in treatment effects (age, gender, intellectual quotient, and comorbidity). No significant differences were detected in relation to interactions between clinical and demographic characteristics of treatment effects in any of the different outcome measures. A nonparametric test for independent samples detected no significant effect of type of medication used on response to DAT intervention.

## Discussion

We predicted that 12 sessions of DAT therapy would result in the following: improvements on social skills, reductions on internalizing and externalizing symptoms, and lower scores on FASD severity.

As our first hypothesis predicted, the DAT group achieved improvements on social skills. Previous studies on DAT showed similar results (Fung and Leung, 2014; Schuck et al., 2015), obtaining gains in this feature according to parent ratings. These results suggest that a DAT offers a novel therapeutic strategy that may enhance traditional evidence-based interventions such as cognitive-behavioral treatments for children and adolescents with FASD.

Our second hypothesis was only partially proved, as reductions on externalizing symptoms were observed but no significant differences were obtained in internalizing symptoms. Previous studies on socials skills training in FASD such as the Friendship Program (O’Connor et al., 2006), demonstrated a correlation between gains on social skills and improvements on child’s behavior. This could also explain the results on externalizing symptoms in our sample. Other previous studies on DAT observed decreases in internalizing symptoms, but no significant differences in relation to externalizing symptoms (Muela et al., 2017). The sample of this previous study was mainly composed of patients with anxiety-depressive disorders, whereas a 76–81% of our sample presented a comorbid ADHD but no internalizing disorders. These differences in the sample characteristics could explain the different results. In spite of this, the comparison of other studies on DAT is difficult because of different diagnosis, different treatment programs and the heterogeneity of measures used.

As predicted by our third hypothesis, severity of FASD symptoms was reduced. Similar results were obtained on adolescents with acute mental disorders, obtaining also improvements on global functioning (Stefanini et al., 2015). Thus, positive effects are not limited to the patient-animal interaction and can be extended to the patient’s global improvement. One possible explanation for this success is the role of the animal as a facilitator of the therapeutic process which can help to create a more relaxed setting.

There are a number of limitations to this study. First, effects of DAT in participants without pharmacological treatment were not assessed. Thus, we can conclude that DAT could be and effective adjunctive treatment but results with DAT alone are unknown. Similarly, most of the previous studies include multimodal and combined treatments (Stefanini et al., 2015). Secondly, our program demonstrated significant short-term gains; however, the evaluation of the maintenance of these achievements is still in progress. Studies on maintenance of the results are needed. Third, we compared an intensive treatment (weekly) combined with pharmacological treatment versus a TAU (monthly), so results have to be interpreted with caution. Furthermore, we do not use self-report measures for these patients, due to the fact that patients with FASD have poor self-awareness and the validity of their self-reports might be unclear (Rasmussen and Bisanz, 2009). Lastly, we applied a structured intervention for children and adolescents. Future studies should adapt treatment modules and modify contents according to patients’ age. The current research is a preliminary pilot study and results must be interpreted with caution due to the small sample size and the low power of the study.

Our research suggests that a DAT intervention in combination to pharmacological treatment could benefit children and adolescents with FASD. It is important to note that the completion rate of the program was high and that low rates of dropout were observed, despite the difficulties of engaging this population. DAT may be especially well suited to people with FASD because animals communicate non-verbally, which may be a less stressful form of interaction than a conversation which requires metacognitive and introspective aspects (Jones et al., 2019). We could hypothesize that animals could act as social catalysts, allowing patients to become more willing to communicate and could facilitate improvements in social interaction and behavioral regulation. Thus, DAT intervention would appear to be an acceptable approach in patients with FASD.

## Data Availability Statement

The datasets generated for this study are available on request to the corresponding author.

## Ethics Statement

The studies involving human participants were reviewed and approved by Clinical Research Ethics Committee Vall d’Hebron Hospital. Written informed consent to participate in this study was provided by the participants’ legal guardian/next of kin.

## Author Contributions

RV led the design, interpretation, and writing of this work. LV participated in the development and implementation of the intervention, the preparation of the data, and contributed to the writing of the manuscript. FR and ED participated in the development and implementation of the intervention. NG-B and JR-Q participated in the design of the project, patient recruitment, and revision of the final manuscript. MS and CV contributed to the development of the project and to the writing of this work.

## Conflict of Interest

The authors declare that the research was conducted in the absence of any commercial or financial relationships that could be construed as a potential conflict of interest.
